# Active ingredients of Astragalus–ginseng against COPD-associated sarcopenia: network pharmacology and molecular dynamics simulations

**DOI:** 10.7717/peerj.21672

**Published:** 2026-08-27

**Authors:** Xiaowen Wu, Jiaxiang Li, Jianan He, Zhiyue Yu, Ming Gao, Limei Geng

**Affiliations:** 1 Graduate School, Hebei University of Traditional Chinese Medicine, Shijiazhuang, China; 2 Department of Respiratory and Critical Care Medicine, The First Affiliated Hospital of Hebei University of Traditional Chinese Medicine, Shijiazhuang, China

**Keywords:** COPD-related sarcopenia, *Astragalus-ginseng*, Network pharmacology, Molecular dynamics simulation, Formononetin, PTGS2

## Abstract

**Background:**

Chronic obstructive pulmonary disease (COPD)-associated sarcopenia is a critical comorbidity associated with high morbidity and mortality, driven by chronic systemic inflammation, oxidative stress, and proteometabolic dysregulation. The *Astragalus membranaceus-Ginseng* (HQ-RS) herbal pair is a traditional remedy for consumptive diseases; however, its molecular mechanisms against this comorbidity remain undefined.

**Methods:**

We integrated transcriptomic data from four Gene Expression Omnibus (GEO) datasets (two discovery cohorts and two validation cohorts) for COPD and sarcopenia. Differentially expressed genes (DEGs) were identified using the thresholds of *P* < 0.05 and —log_2_FC— ≥ 1. Active compounds of HQ-RS were screened using the Traditional Chinese Medicine Systems Pharmacology Database and Analysis Platform (TCMSP) database (oral bioavailability (OB) ≥ 30%, drug-likeness (DL) ≥ 0.18). Candidate targets were identified by intersecting drug targets with shared disease targets (GeneCards relevance score > 20). A protein-protein interaction (PPI) network was constructed to identify hub genes using multi-centrality metrics. Molecular docking was performed with celecoxib as a positive control. Three independent 100 ns molecular dynamics (MD) simulations of the FMNT-PTGS2 complex were then performed, and the trajectories were analyzed using the Simulation Interaction Diagram (SID) tool.

**Results:**

We identified 1,267 COPD-specific and 115 sarcopenia-specific DEGs, yielding nine shared DEGs. Intersection of drug targets with 279 shared disease targets identified 36 candidate targets. PPI network analysis highlighted PTGS2, CDKN1A, NFE2L2, NOS3, and MMP2 as key hub genes. Molecular docking revealed that formononetin (FMNT) exhibited the most favorable binding affinity to PTGS2 (−8.94 kcal/mol), comparable to the positive control celecoxib (−7.97 kcal/mol). Three independent 100 ns MD simulations of the FMNT-PTGS2 complex demonstrated that the system reached dynamic equilibrium after approximately 20 ns in all replicates, with the ligand maintaining a stable binding pose throughout the simulations. Molecular Mechanics Generalized Born Surface Area (MM-GBSA) calculations across three replicate simulations yielded a mean binding free energy of −47.82 ± 1.13 kcal/mol, supporting the thermodynamic favorability of the interaction. Protein-ligand interaction analysis identified sustained hydrogen bonds and hydrophobic contacts with key active-site residues.

**Conclusion:**

This study computationally predicts that the FMNT-PTGS2 interaction may represent a potential mechanism underlying the efficacy of the HQ-RS herbal pair in treating COPD-associated sarcopenia. This study is entirely computational and limited by small discovery cohort sample sizes (COPD *n* = 6, sarcopenia *n* = 51). These findings provide a multi-component, multi-target mechanistic hypothesis that requires experimental validation.

## Introduction

Chronic obstructive pulmonary disease (COPD) is a prevalent respiratory disorder characterized by persistent respiratory symptoms and irreversible airflow limitation (Sullivan et al., 2018; Xu et al., 2024). According to the 2023 Global Burden of Disease Study, COPD affects more than 380 million people worldwide and remains the third leading cause of death, placing a substantial burden on health systems and the broader socioeconomic environment, particularly in low- and middle-income countries (GBD 2023 Asia Chronic Respiratory Disease Collaborators, 2026). This disease constitutes a significant global health burden characterized by high morbidity and mortality (Halpin et al., 2019). Sarcopenia, a progressive skeletal muscle disorder characterized by loss of muscle mass and function, is a common and debilitating comorbidity in COPD patients, with a prevalence ranging from 15% to 55% (Cruz-Jentoft et al., 2019). The combination of COPD and sarcopenia is particularly concerning. Epidemiological studies have indicated that patients with both conditions face a 2- to 3-fold higher 5-year mortality rate and increased rates of hospitalization (Jones et al., 2015; Sepúlveda-Loyola et al., 2020), creating a vicious cycle that severely impairs patients’ quality of life.

The interaction between COPD and sarcopenia involves a complex network of pathological mechanisms and forms a self-reinforcing vicious cycle. Beyond physical deconditioning, this comorbidity is driven by shared molecular mechanisms characterized by chronic systemic inflammation and metabolic dysregulation. Specifically, sustained elevation of pro-inflammatory cytokines (notably TNF-α and IL-6) activates the ubiquitin-proteasome system (UPS) and autophagy-lysosome pathways, accelerating muscle protein catabolism (Adami et al., 2026). Concurrently, hypoxia-induced oxidative stress and corticosteroid therapy impair mitochondrial function and insulin signaling, thereby suppressing the PI3K-Akt/mTOR pathway and inhibiting muscle protein synthesis (Benz et al., 2019; Benz et al., 2023). This dual disruption—excessive degradation coupled with suppressed synthesis—leads to substantial muscle wasting and respiratory muscle weakness. Consequently, patients with COPD-associated sarcopenia exhibit reduced cough efficacy, heightened infection risk, diminished exercise tolerance, and rapid deterioration in lung function and quality of life (Kaluźniak-Szymanowska et al., 2021). Epidemiological data further suggest that, compared with patients without sarcopenia, those with sarcopenia experience a two- to three-fold higher frequency of exacerbations, rehospitalizations, and all-cause mortality (He et al., 2023). In COPD, hypoxia and persistent inflammation promote muscle catabolism; conversely, respiratory muscle weakness resulting from sarcopenia exacerbates pulmonary symptoms (Yu et al., 2024). Despite the clinical significance, comprehensive therapeutic strategies addressing both conditions concurrently are limited.

The *Astragalus membranaceus-ginseng* (HQ-RS) herb pair, widely used in traditional medicine, has shown potential in the management of chronic diseases (Tran, Kim & Lee, 2024). Both herbs are recognized for their adaptogenic and health-promoting properties. Although formononetin (FMNT) and kaempferol are widely distributed in plants, their relevance is particularly pronounced in the context of the HQ-RS herbal pair. FMNT is a major isoflavone in *Astragalus membranaceus*, while kaempferol and its glycosides are well-documented components of both *Astragalus* and *ginseng*, underpinning their traditional pharmacological activities (Liu et al., 2025a; Luo et al., 2025). Extensive preclinical studies have demonstrated that FMNT exhibits anti-inflammatory, antioxidant, and anti-fibrotic properties through multiple signaling pathways, including Nrf2/HO-1, NF-κB, PI3K/Akt, ERK, and the NLRP3 inflammasome (Yan & Wang, 2025). Consequently, FMNT has been investigated in various progressive diseases, and recent advances in targeted nano-delivery systems have further expanded its therapeutic potential (Ouyang et al., 2023; Liu et al., 2025b). However, research on FMNT in specific pulmonary diseases, such as COPD, remains limited. Moreover, the role of FMNT in sarcopenia has only been speculated upon, and no study has yet explored its therapeutic effect on COPD-associated sarcopenia (Fang, Wei & Lei, 2025). This knowledge gap underscores the need for a systematic investigation into whether FMNT, as a core active component of HQ-RS, could simultaneously target the shared pathological mechanisms of COPD and sarcopenia. Specifically, no study has (i) identified shared transcriptomic signatures between COPD and sarcopenia using integrated Gene Expression Omnibus (GEO) datasets, (ii) systematically predicted which HQ-RS compounds could simultaneously target these shared pathways, or (iii) evaluated the binding stability of the predicted compound-target pair using molecular dynamics (MD) simulation.

Modern pharmacological studies have revealed that both herbs and their combination (HQ-RS) exhibit various bioactivities, including immunomodulatory, antioxidant, anti-inflammatory, and myoprotective effects (Chu et al., 2016; Bilia & Bergonzi, 2020; Zhang et al., 2023; Chen et al., 2025). For instance, HQ-RS administration has been associated with improved exercise capacity in COPD patients (Zhang et al., 2023), and ginseng extracts have demonstrated anti-inflammatory and antioxidant properties relevant to COPD-associated complications (Yeh et al., 2022; Bagherniya et al., 2022; Zha et al., 2022; Ding et al., 2023). However, the specific active compounds, molecular targets, and integrated pathways through which HQ-RS exerts its therapeutic effects on COPD-associated sarcopenia remain poorly defined. The emergence of computational biology techniques—such as network pharmacology, molecular docking, and molecular dynamics (MD) simulation—offers a powerful paradigm for deconvoluting the complex mechanisms of multicomponent herbal medicines (Wang et al., 2022; Xu et al., 2025; Dorjay Tamang et al., 2025). Therefore, this study aimed to leverage these integrated approaches to systematically predict the active components, core targets, and key signaling pathways of HQ-RS against this comorbidity (Pinzi & Rastelli, 2019).

## Materials & Methods

### Data sources and preprocessing

#### GEO transcriptomic datasets

Gene Expression Omnibus (GEO, http://www.ncbi.nlm.nih.gov/geo/) datasets were categorized by disease type and usage into discovery and validation cohorts. For COPD, GSE29133 (three COPD *vs.* three healthy controls, human distal lung/alveolar epithelial type II cells) served as the discovery cohort, and GSE38974 (23 COPD *vs.* nine controls, human lung tissue) as the validation cohort. For sarcopenia, GSE8479 (25 sarcopenia *vs.* 26 controls, human skeletal muscle/vastus lateralis) was used as the discovery cohort, and GSE1428 (12 sarcopenia *vs.* 10 controls, human skeletal muscle/vastus lateralis) as the validation cohort. Sample compositions, sizes, and tissue sources are summarized in Table 1.

#### Transcriptomic preprocessing and differential expression analysis

Raw expression matrices from GEO were downloaded and mapped to gene symbols according to the platform annotation files. When multiple probes corresponded to the same gene, the median expression value was retained. For datasets where the maximum raw expression value exceeded 100, log_2_(x+1) transformation was applied, followed by quantile normalization using the *normalizeBetweenArrays* function in the *limma* R package (Ritchie et al., 2015). Batch effects across platforms were not corrected due to the distinct tissue sources (lung *vs.* skeletal muscle); instead, the discovery and validation cohorts were analyzed independently to avoid cross-tissue confounding.

Differential expression analysis was performed using a linear model implemented in limma, with the design matrix comparing disease *versus* control groups (Davis & Meltzer, 2007). Empirical Bayes moderation (eBayes) was applied for statistical inference. Differentially expressed genes (DEGs) were identified using a threshold of *P*-value < 0.05 and —log_2_FC— ≥ 1. The volcano plot of differentially expressed genes (DEGs) in the discovery set and validation set, as well as the heatmap of the top 50 significantly differentially expressed genes (DEGs), can be found in Figs. S1 and S2.

We acknowledge that this permissive *P*-value cutoff was deliberately chosen to maximize sensitivity given the inherently small sample sizes of the discovery cohorts (COPD: *n* = 6; sarcopenia: *n* = 51). Although this threshold may increase the false positive rate, the identified candidate DEGs were subsequently evaluated for expression direction consistency in independent validation cohorts to mitigate this limitation.

**Table 1:** Summary of GEO datasets used in this study.

**Dataset_ID**	**Purpose**	**Sample_composition**	**Sample_size**	**Tissue_source**
GSE29133	Discovery	3 disease + 3 control	6	Human distal lung; alveolar epithelial type II cells
GSE38974	Validation	23 disease + 9 control	32	Human lung tissue
GSE8479	Discovery	25 disease + 26 control	51	Human skeletal muscle; vastus lateralis
GSE1428	Validation	12 disease + 10 control	22	Human skeletal muscle; vastus lateralis

#### Discovery-validation consistency assessment

Consistency between the discovery and validation cohorts was assessed by merging gene symbols and comparing the direction of log_2_FC. Scatter plots were generated with the discovery log_2_FC on the x-axis and the validation log_2_FC on the y-axis, including all DEGs from the discovery cohort that could be mapped to the validation cohort. Genes with consistent direction of change that also met the significance threshold (*P* < 0.05, —log_2_FC— ≥ 1) in the validation cohort were highlighted in red, with the proportion of validated genes annotated on the plot. For the nine shared DEGs, directional consistency between discovery and validation datasets was assessed separately, as these genes are of primary interest for downstream analysis.

### Network pharmacology

#### Screening of active compounds and targets

Active compounds of *Astragalus membranaceus* (Huangqi) and *Panax ginseng* (Renshen) were retrieved from the Traditional Chinese Medicine Systems Pharmacology Database and Analysis Platform (TCMSP, https://www.tcmsp-e.com/tcmsp.php). Screening criteria were oral bioavailability (OB) ≥ 30% and drug-likeness (DL) ≥ 0.18 (Ru et al., 2014). These thresholds are widely adopted in network pharmacology studies but are empirical rather than biologically derived (Zhang, Yuan & Huang, 2022; Ko et al., 2022). We acknowledge that this approach may exclude compounds with low predicted oral bioavailability but potential local or intravenous efficacy. The full list of 41 compounds meeting these criteria is provided in Table S1. Target genes corresponding to each compound were obtained from the TCMSP target mapping table and standardized using the UniProt database (https://www.uniprot.org/) for gene symbol conversion. Disease targets for COPD and sarcopenia were integrated from the GeneCards (https://www.genecards.org) (Bai et al., 2021), OMIM (https://www.omim.org) (Amberger et al., 2015), and CTD (https://ctdbase.org/) databases. For GeneCards, a relevance score threshold of >20 was applied to refine the target list to those most relevant to the disease. After merging and deduplication, 3,599 COPD targets and 465 sarcopenia targets were obtained, yielding 279 shared targets between the two diseases.

#### Disease-drug target integration

Disease targets were integrated to obtain COPD-specific targets, sarcopenia-specific targets, and shared targets between the two diseases. Drug targets were based on the TCMSP target mapping table, standardized using UniProt/gene symbol conversion, and deduplicated. Final drug-disease candidate targets were obtained by intersecting the drug targets with the shared disease targets, incorporating candidate DEG evidence and prioritized pharmacological data.

#### STRING PPI network and hub gene identification

The 36 candidate drug-disease targets were imported into the STRING database (https://string-db.org/) (Von Mering et al., 2003) with the species set to *Homo sapiens* and a minimum required interaction confidence score of 0.4. The resulting protein-protein interaction (PPI) network was visualized using Cytoscape (v3.9.1) (Shannon et al., 2003).

Network centrality analysis was performed using the CytoHubba plugin (Nangraj et al., 2020). Four centrality metrics were calculated: Degree, Betweenness, Closeness, and Maximal Clique Centrality (MCC). To identify core hub genes, we employed a multi-centrality comprehensive assessment method: genes ranking in the top 20 for at least three out of four centrality metrics were designated as core hub genes. This approach provides a more robust selection than relying on a single centrality metric and reduces the influence of metric-specific biases.

#### Functional enrichment analysis

Gene Ontology (GO) and Kyoto Encyclopedia of Genes and Genomes (KEGG) enrichment analyses were performed using the clusterProfiler R package (Wu et al., 2021). Gene IDs were converted from SYMBOL to ENTREZID using org.Hs.eg.db. GO analysis covered Biological Process (BP), Cellular Component (CC), and Molecular Function (MF) ontologies. *P*-values were adjusted using the Benjamini–Hochberg (BH) method, and an adjusted *P*-value < 0.05 was considered statistically significant. KEGG analysis was performed with the species parameter set to “hsa”. To address pathway redundancy among GO terms, the simplify function in clusterProfiler was applied with a similarity cutoff of 0.7 to remove highly similar terms, retaining only the most representative pathways for interpretation.

### Molecular docking

#### Preparation of ligands and receptors

Candidate compound-target pairs were selected from the drug-disease candidate targets and core database targets. Receptor protein structures were obtained from AlphaFold, and ligand 3D structures were retrieved from the PubChem database (https://pubchem.ncbi.nlm.nih.gov/) and saved in SDF format. The active compounds were converted to three-dimensional structures using Chem3D 23.1.1. PDBQT conversion was performed using Meeko/OpenBabel.

#### Docking protocol

Molecular docking was performed using AutoDock Vina. Docking grid boxes were centered on the receptor coordinates with a box size of up to 38 Å. Binding energies (kcal/mol) were recorded and log files were retained. To assess the docking protocol, we included celecoxib, a known PTGS2 (COX-2) inhibitor, as a positive control. Docking results provide predicted binding affinities and are not a substitute for experimental validation. All docking predictions require experimental confirmation.

#### Docking visualization

Docking visualization was performed using PyMOL (v3.1). Green dashed lines indicate polar contacts, and magenta dashed lines indicate hydrophobic contacts. Local interaction diagrams and overview diagrams were generated.

### Molecular dynamics simulation

#### System setup

The FMNT-PTGS2 complex was simulated using the Schrödinger 2024 suite (Desmond module, Maestro interface). The initial complex structure was obtained from molecular docking. The system was solvated in an orthorhombic box of TIP3P water molecules with a 10 Åbuffer distance. The total system contained 58,306 atoms, including 16,472 water molecules. One chloride ion (Cl^−^) was added to neutralize the system charge. The Optimized Potentials for Liquid Simulations 3e (OPLS3e) force field was used for its accuracy in describing protein-ligand interactions.

#### Simulation protocol

Energy minimization was performed using the steepest descent algorithm (5,000 steps) followed by the conjugate gradient algorithm (2,000 steps). The system was gradually heated from 0 to 300 K under the canonical (NVT) ensemble, followed by density equilibration under the isothermal–isobaric (NPT) ensemble (1 bar, 300 K). Three independent production MD simulations (Runs 1-3) were conducted for 100.1 ns each with different initial velocity seeds, using a 2 fs time step at 300 K and 1 atm under the NPT ensemble. Temperature was maintained using the Nosé-Hoover chain thermostat with a relaxation time of 1.0 ps, and pressure was maintained using the Martyna-Tobias-Klein barostat with a relaxation time of 2.0 ps.

To assess the internal consistency of each trajectory, each simulation was divided into two 50 ns windows (0–50 ns and 50–100 ns), and key structural metrics (protein backbone root mean square deviation (RMSD), ligand RMSD, and residue-level root mean square fluctuation (RMSF) were compared between the two windows. Structural metrics were averaged across the three replicates using the Simulation Interaction Diagram (SID) tool’s default “Average over replicates” function, and the averaged results are presented in the main text.

#### Trajectory analysis

Trajectories from all three replicate runs were loaded into the Schrödinger Simulation Interaction Diagram (SID) tool for analysis. It should be noted that the Schrödinger Simulation Interaction Diagram (SID) tool applies a default “Average over replicates” function when multiple trajectory files are loaded simultaneously. This function computes and displays the mean structural metrics (RMSD, RMSF, contact fractions) averaged across the three replicates. The following metrics were calculated from the averaged data. Protein backbone RMSD and ligand RMSD (relative to the initial frame) were calculated to assess structural convergence. Cα RMSF and ligand heavy-atom RMSF were calculated to evaluate residue-level and atomic flexibility. Protein-ligand contact analysis: hydrogen bonds, hydrophobic contacts, ionic interactions, and water bridges, with interaction fractions normalized over the trajectory. Ligand conformational properties: RMSD, radius of gyration (rGyr), molecular surface area (MolSA), solvent-accessible surface area (SASA), and polar surface area (PSA). Binding free energy estimation: the binding free energy of the FMNT-PTGS2 complex was calculated using the Prime Molecular Mechanics Generalized Born Surface Area (MM-GBSA) module (Schrödinger, 2024, VSGB 2.0 solvation model, OPLS3e force field). For the MM-GBSA calculation, 50 equally spaced frames from the last 50 ns of each replicate trajectory were extracted. The binding free energy (ΔGbind) was calculated for each frame as: ΔGbind = Gcomplex −(Gprotein + Gligand). The mean and standard deviation across all three replicates are reported. The binding free energy was decomposed into individual energy components: electrostatic interaction (ΔEele), van der Waals interaction (ΔEvdw), non-polar solvation free energy (ΔGnonp), and polar solvation free energy (ΔGsolv GB), where ΔGbind ≈ ΔEvdw + ΔEele + ΔGsolv GB + ΔGnonp.

## Results

### Identification of shared DEGs and validation

**Figure 1:**
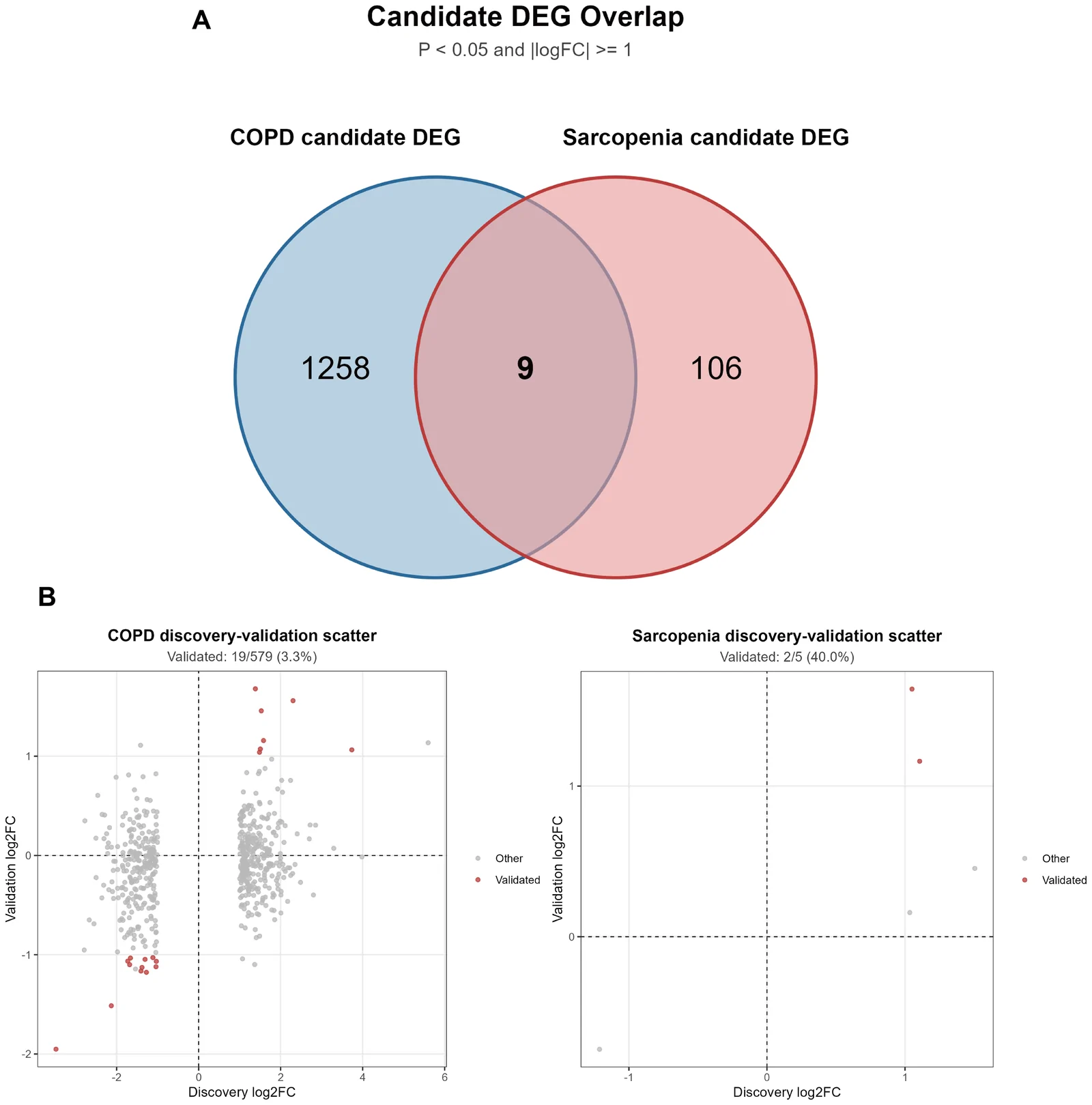
Overview of DEGs and shared genes. (A) Venn diagram of COPD discovery DEGs (1,267 DEGs, of which 1,258 are unique to COPD) and sarcopenia discovery DEGs (115 DEGs, of which 106 are unique to sarcopenia); the intersection contains nine shared genes. (B) Scatter plot of log_2_FC between discovery and validation for COPD (left) and sarcopenia (right). Each point represents a discovery DEG mapped to the validation cohort. Red points indicate genes with consistent expression direction that also met the significance threshold (*P* < 0.05, —log_2_FC— ≥ 1) in the validation cohort; numbers indicate validated DEGs: 19 of 579 mappable genes (3.3%) for COPD and two of five mappable genes (40.0%) for sarcopenia. These validation rates reflect the statistical challenges of cross-tissue, cross-dataset validation. The nine shared DEGs identified in this study were evaluated separately for directional consistency (see main text).

In the discovery cohorts, we identified 1,267 DEGs in COPD and 115 DEGs in sarcopenia using a threshold of *P* < 0.05 and —log_2_FC— ≥ 1. Intersection analysis revealed 9 shared DEGs between the two diseases (Fig. 1A). These shared genes included PTGS2, CDKN1A, NFE2L2, NOS3, MMP2, ADRB2, CYP1B1, ERBB3, and RB1.

To assess the robustness of these findings, we evaluated the expression direction consistency of the nine shared genes in independent validation cohorts (GSE38974 for COPD, GSE1428 for sarcopenia). For context, the validation rate for all discovery DEGs that could be mapped to the validation cohorts was limited −19 of 579 mappable genes (3.3%) for COPD and two of five mappable genes (40.0%) for sarcopenia (Fig. 1B)—and the overlap of DEGs meeting strict thresholds in both cohorts was zero (Fig. S3A), reflecting the statistical challenges of cross-tissue, cross-dataset validation with small sample sizes. Despite these limitations, the expression direction (up- or down-regulation) of the nine shared genes showed high consistency between the discovery and validation datasets. For example, PTGS2 was consistently upregulated in both the COPD discovery and validation cohorts as well as in sarcopenia discovery and validation cohorts. This directional concordance across independent datasets, despite differences in tissue source (lung *vs.* skeletal muscle) and sample size, supports the biological relevance of these nine candidate genes for COPD-associated sarcopenia. We acknowledge that this directional consistency alone does not confirm their functional significance; rather, it provides a computationally prioritized set of targets for further analysis.

### Network pharmacology analysis

#### Active compounds and targets

From the TCMSP database, 41 active compounds from *Astragalus membranaceus* and *Panax ginseng* met the screening criteria (OB ≥ 30%, DL ≥ 0.18). The top 12 compounds ranked by degree (connectivity in the compound-target network) are listed in Table 2. Key compounds included quercetin (degree = 153), kaempferol (124), 7-O-methylisomucronulatol (45), FMNT (39), and isorhamnetin (37). Mapping these compounds to their target genes through the TCMSP-UniProt pipeline yielded 224 unique target genes.

**Table 2:** Top active compounds of HQ-RS.

**Compound**	**OB_percent**	**DL**	**Degree**	**Source_herb**
quercetin	46.433	0.275	153	*Astragalus membranaceus*
kaempferol	41.882	0.241	124	*Ginseng& Astragalus membranaceus*
7-O-methylisomucronulatol	74.686	0.298	45	*Astragalus membranaceus*
formononetin	69.674	0.212	39	*Astragalus membranaceus*
isorhamnetin	49.604	0.306	37	*Astragalus membranaceus*
3,9-di-O-methylnissolin	53.742	0.476	23	*Astragalus membranaceus*
hederagenin	36.914	0.751	23	*Astragalus membranaceus*
(6aR,11aR)-9,10-dimethoxy-6a,11a-dihydro-6H-benzofurano[3,2-c]chromen-3-ol	64.255	0.425	22	*Astragalus membranaceus*
Calycosin	47.752	0.243	22	*Astragalus membranaceus*
Jaranol	50.829	0.291	13	*Astragalus membranaceus*
Bifendate	31.098	0.666	7	*Astragalus membranaceus*
1,7-Dihydroxy-3,9-dimethoxy pterocarpene	39.045	0.479	4	*Astragalus membranaceus*

#### Drug-disease candidate targets

**Figure 2:**
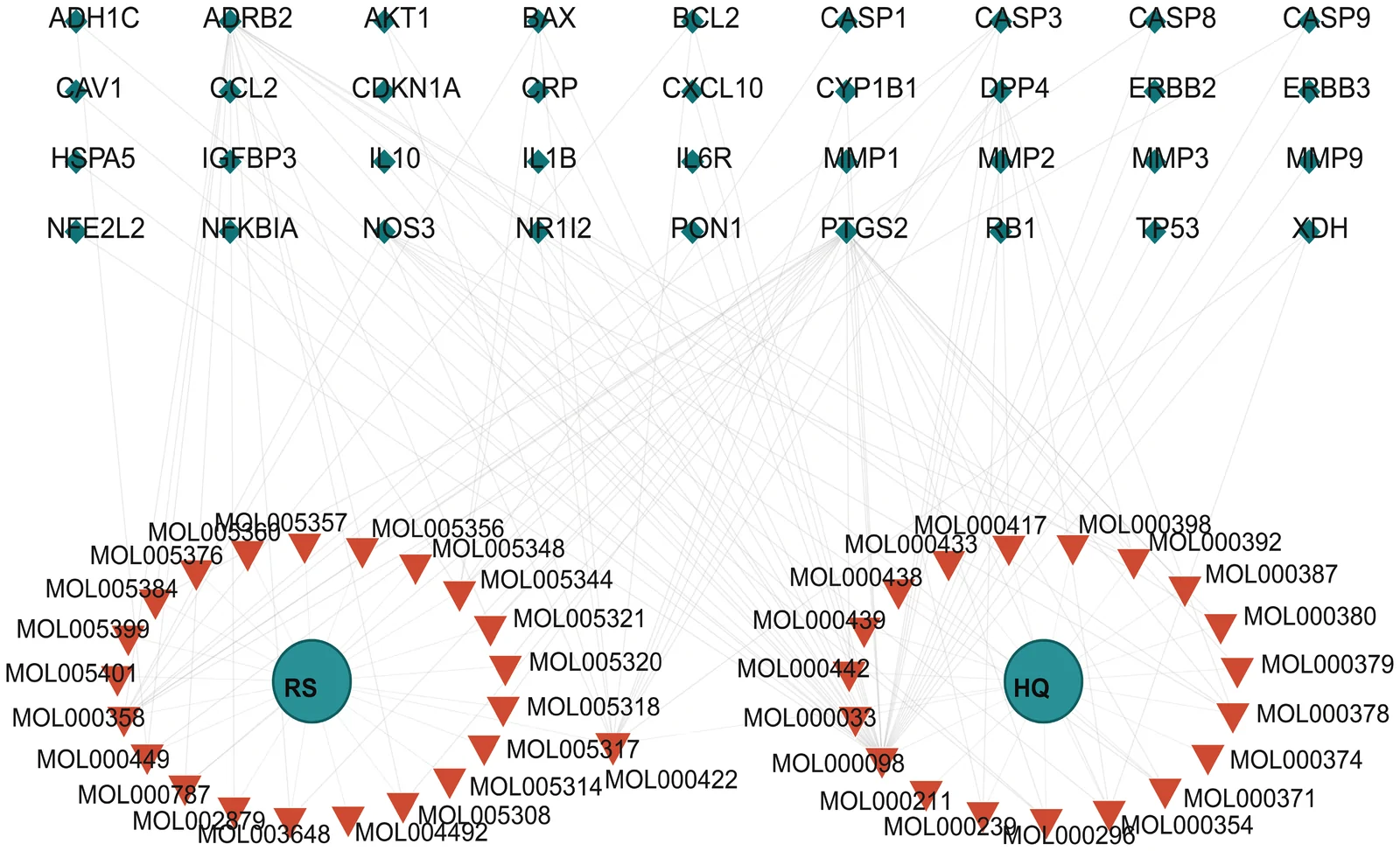
Herb-compound-target network. Green circular nodes represent the two herbs (*Astragalus membranaceus* and *Panax ginseng*). Red nodes represent active compounds; green square nodes represent target proteins. Edges indicate compound-target interactions. Core hub targets (NOS3, PTGS2, MMP2, ADRB2) are labeled.

Disease target databases provided 3,599 COPD targets and 465 sarcopenia targets. Intersection of these two disease target sets identified 279 shared disease targets potentially involved in the pathological mechanisms common to both conditions. The drug targets (224) were then intersected with these 279 shared disease targets, yielding 36 candidate drug-disease targets (Fig. S3B). These 36 targets represent the molecular intersection where HQ-RS compounds may act upon the shared pathological mechanisms of COPD and sarcopenia. These targets were used for all subsequent analyses. The herb-compound-target network is shown in Fig. 2.

#### Hub gene identification *via* PPI network

**Figure 3:**
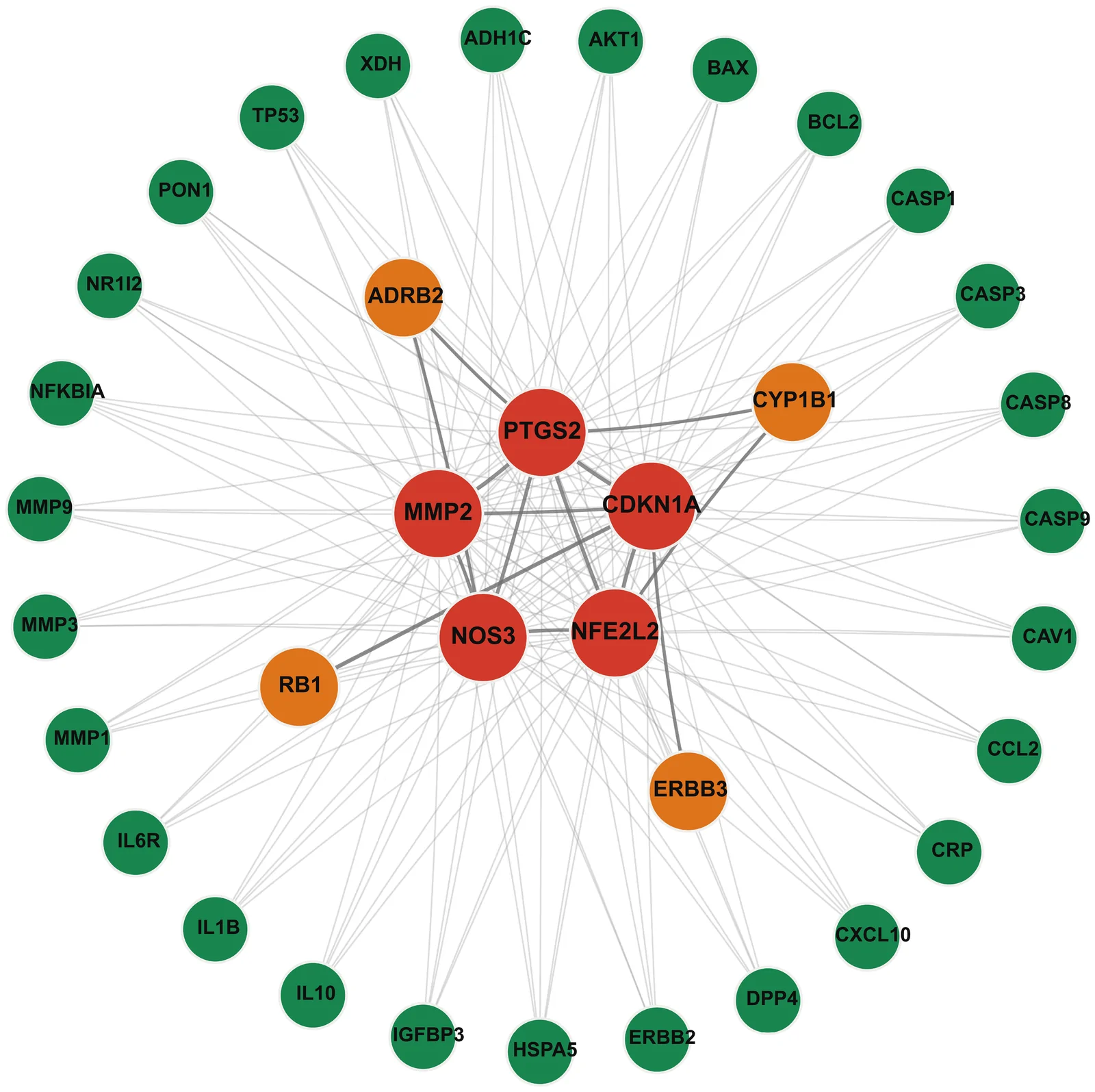
PPI network of core hub genes. Nodes represent candidate targets; node size reflects degree (larger = higher connectivity). Red nodes: core hub genes (*PTGS2, CDKN1A, NFE2L2, NOS3, MMP2*). Orange and green nodes: other candidate targets. Edges represent protein-protein interactions (confidence > 0.4). The network contains 36 nodes and 170 edges.

The 36 candidate targets were imported into the STRING database (confidence score > 0.4) to construct a PPI network. The resulting network contained 36 nodes and 14 edges. Using CytoHubba with four centrality metrics (Degree, Betweenness, Closeness, MCC), we applied a multi-centrality comprehensive assessment to identify core hub genes. Five core hub genes were identified that ranked in the top 20 for at least three of the four metrics: PTGS2, CDKN1A, NFE2L2, NOS3, and MMP2 (Table 3). PTGS2 exhibited the highest degree (6) and ranked first across all four metrics, indicating its central role in the network. The PPI network is shown in Fig. 3.

#### Pathway enrichment analysis

**Figure 4:**
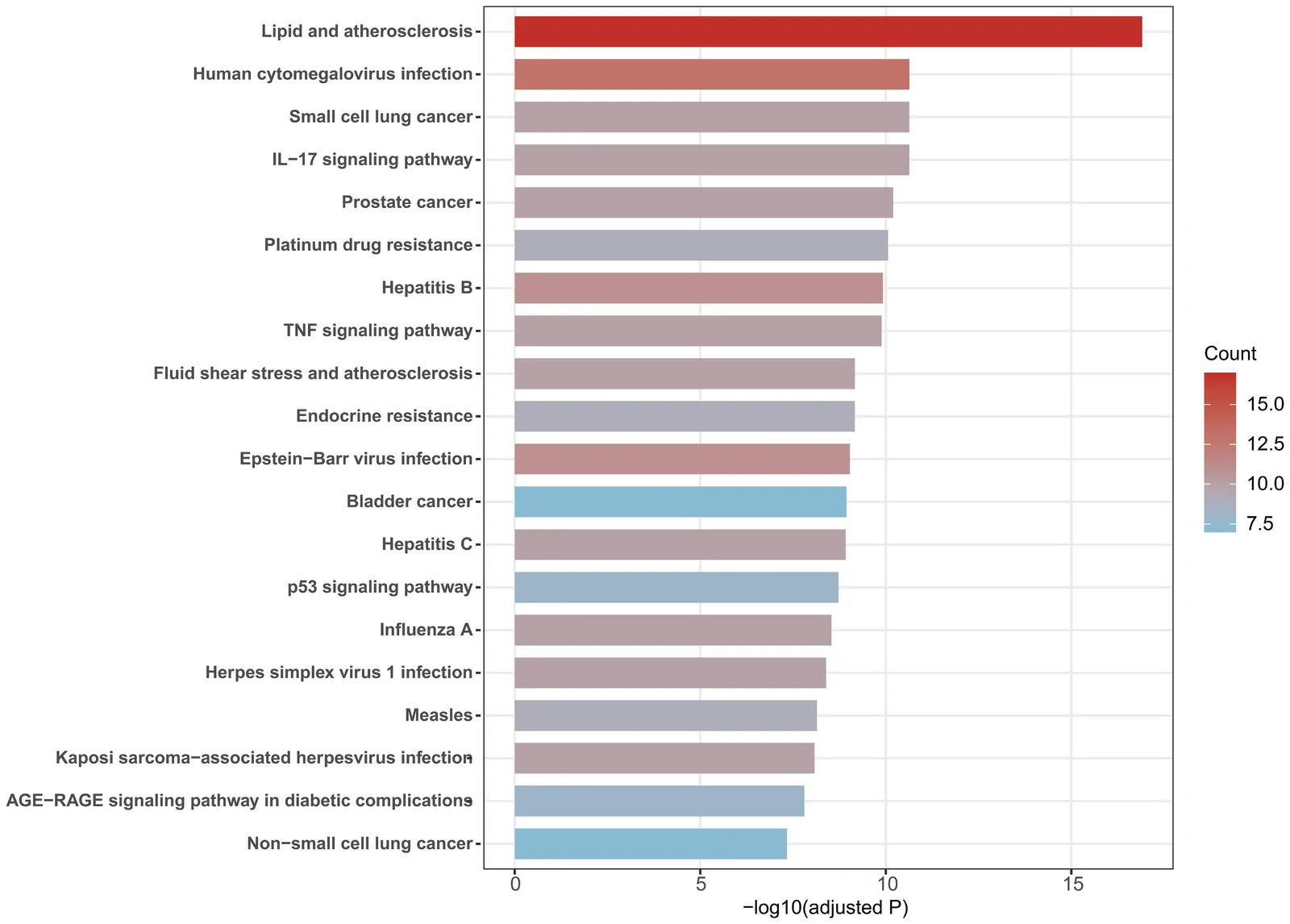
KEGG pathway enrichment bar plot (top 20 significant pathways). The *x*-axis shows −log_10_ (adjusted *P*-value). Bars are colored by significance. Key pathways include: lipid and atherosclerosis, IL-17, TNF, p53, AGE-RAGE, and several infection-related pathways.

Functional enrichment analysis of the 36 candidate targets identified 112 significantly enriched KEGG pathways (adjusted *P* < 0.05, BH correction). The top 20 pathways (Fig. 4) were predominantly associated with inflammatory signaling (IL-17, TNF), metabolic stress (AGE-RAGE), and cellular stress responses (p53, FoxO). Additional enriched pathways included fluid shear stress and atherosclerosis, and pathways related to infectious diseases and cancer. These results indicate that HQ-RS may exert its effects by concurrently modulating inflammatory, metabolic, and stress-responsive pathways relevant to both COPD and sarcopenia.

**Table 3:** Multi-centrality rankings of candidate targets.

**Gene**	**Degree**	**Betweenness_rank**	**Closeness_rank**	**MCC_rank**
PTGS2	6	2	1	1
CDKN1A	5	1	2	2
NFE2L2	4	3	3	2
NOS3	4	4	5	2
MMP2	3	5	4	5

### Molecular docking results

Molecular docking was performed between the top active compounds and the five core hub genes, yielding 15 compound-target pairs. Predicted binding energies ranged from −8.94 to −7.98 kcal/mol (all below −7.0 kcal/mol, indicating favorable predicted affinity). The top five docking pairs are listed in Table 4.

**Figure 5:**
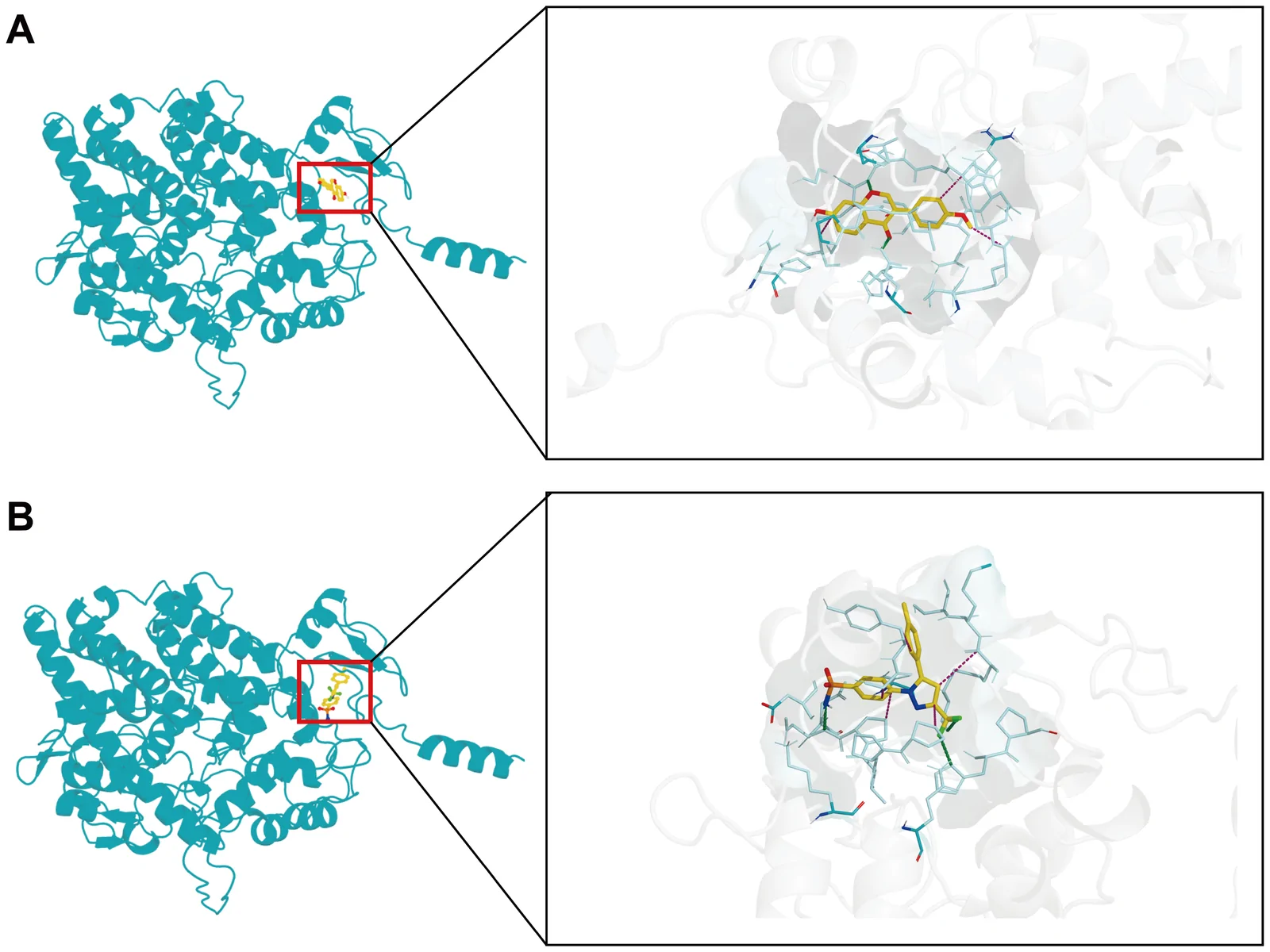
Molecular docking results. (A) Overview and close-up view of FMNT bound to PTGS2 and (B) celecoxib bound to PTGS2. Green dashed lines: hydrogen bonds; magenta dashed lines: hydrophobic contacts.

FMNT exhibited the most favorable binding affinity to PTGS2 (−8.94 kcal/mol) (Fig. 5A), which was stronger than that of the positive control celecoxib (−7.97 kcal/mol) (Fig. 5B), a well-characterized PTGS2 (COX-2) inhibitor included to benchmark our docking protocol. Other strong interactions included β-sitosterol-NOS3 (−8.93 kcal/mol), quercetin-MMP2 (−8.62 kcal/mol), quercetin-RB1 (−8.45 kcal/mol), and quercetin-NOS3 (−8.34 kcal/mol). Docking visualization revealed that FMNT formed stable polar and hydrophobic contacts within the PTGS2 binding pocket.

It is important to note that PTGS2 is a promiscuous target that binds a wide range of flavonoid compounds. Therefore, the favorable docking scores predict a potential interaction but do not confirm selective or physiologically relevant inhibition; this requires dedicated experimental validation. The FMNT-PTGS2 pair is prioritized as a computationally predicted compound-target candidate for subsequent experimental investigation.

### Molecular dynamics simulations of the FMNT-PTGS2 complex

Based on the docking results, the FMNT-PTGS2 complex was selected for MD simulation due to its highest binding affinity.

**Table 4:** Top five molecular docking results.

**Molecule name**	**Gene**	**Affinity (kcal/mol)**
formononetin	PTGS2	−8.936
beta-sitosterol	NOS3	−8.928
quercetin	MMP2	−8.624
quercetin	RB1	−8.450
quercetin	NOS3	−8.337
celecoxib	PTGS2	−7.969

#### System stability and convergence

**Figure 6:**
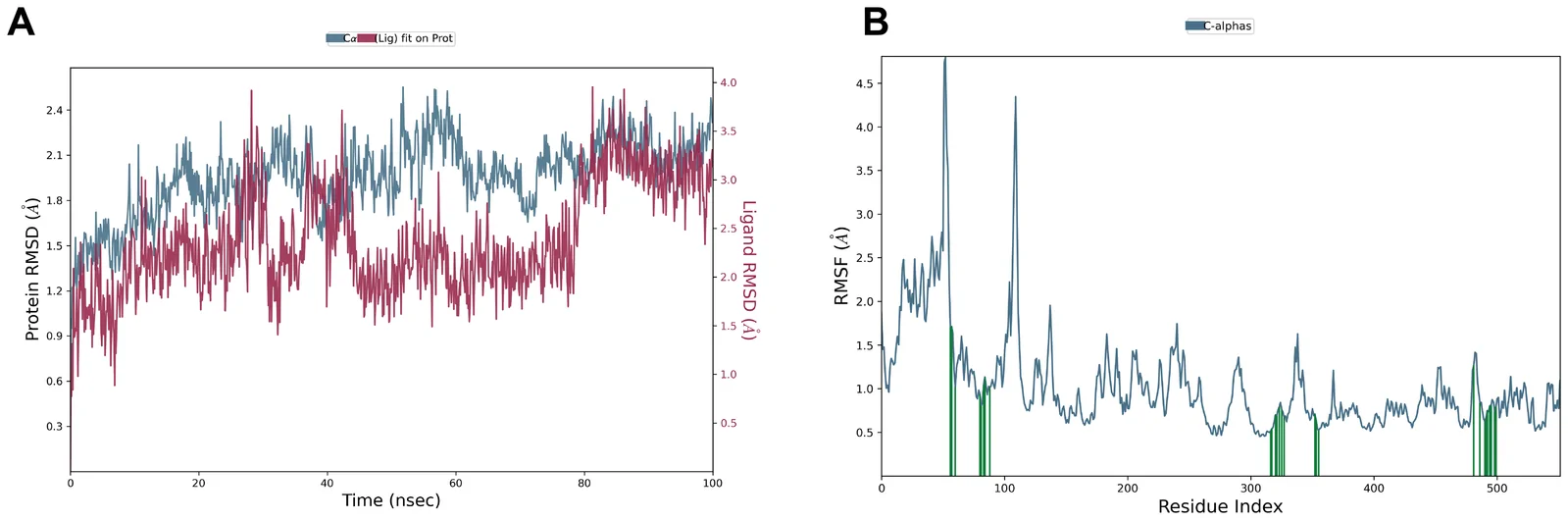
Molecular dynamics simulation of the PTGS2-FMNT complex. (A) RMSD evolution of the protein backbone (left *Y*-axis) and ligand heavy atoms (right *Y*-axis) over 100 ns, averaged across three independent replicate simulations. (B) RMSF of PTGS2 Cα atoms and FMNT heavy atoms, averaged across three replicates.

Three independent 100 ns MD simulations (Runs 1-3) were performed with different initial velocity seeds to evaluate the dynamic stability and reproducibility of the FMNT-PTGS2 binding complex. The trajectories were analyzed using the Schrödinger SID tool, which applies a default “Average over replicates” function; consequently, the structural metrics presented below represent the mean values averaged across the three replicates. The protein backbone RMSD (averaged across three replicates) increased rapidly during the first ∼20 ns and subsequently stabilized around a plateau value of approximately 2.2 Å for the remaining 80 ns (Fig. 6A), with fluctuations remaining within 1.0 Å over the last 50 ns (mean RMSD over the last 50 ns: 2.1 ± 0.2 Å), indicating that the tertiary structure of PTGS2 was well preserved throughout all simulations. The ligand RMSD (aligned on the protein backbone) remained relatively low (typically <1.5 Å after the initial equilibration phase), with a mean value of approximately 1.0 Å, suggesting that FMNT maintained a consistent binding pose within the active site without major repositioning events.

To assess the internal consistency of the trajectories, key metrics were compared between the first and second 50 ns windows for each replicate. The mean protein backbone RMSD across replicates was 2.0 ± 0.4 Å (0–50 ns) and 2.3 ± 0.2 Å (50–100 ns); the mean ligand RMSD was 0.9 ± 0.2 Å and 1.1 ± 0.3 Å, respectively. This modest increase reflects continued sampling of conformational space around a stable binding mode, rather than system instability. RMSF analysis (Fig. 6B) showed that active-site residues exhibited lower fluctuations (Cα RMSF typically <1.2 Å), whereas distal loop regions showed higher flexibility, as expected. Ligand RMSF analysis revealed that the core scaffold atoms (atoms #5-9, 12, 15, 19, 20) exhibited low RMSF values (0.14−0.23 Å), indicative of rigid binding, while terminal functional groups (atoms #13, 14, 17, 18) showed higher RMSF values (0.7−1.2 Å), reflecting greater conformational flexibility. Due to the SID tool’s default “Average over replicates” function, the RMSF values presented represent the mean across the three replicates, and replicate-specific variability could not be assessed from this output.

#### Protein-ligand interaction analysis

**Figure 7:**
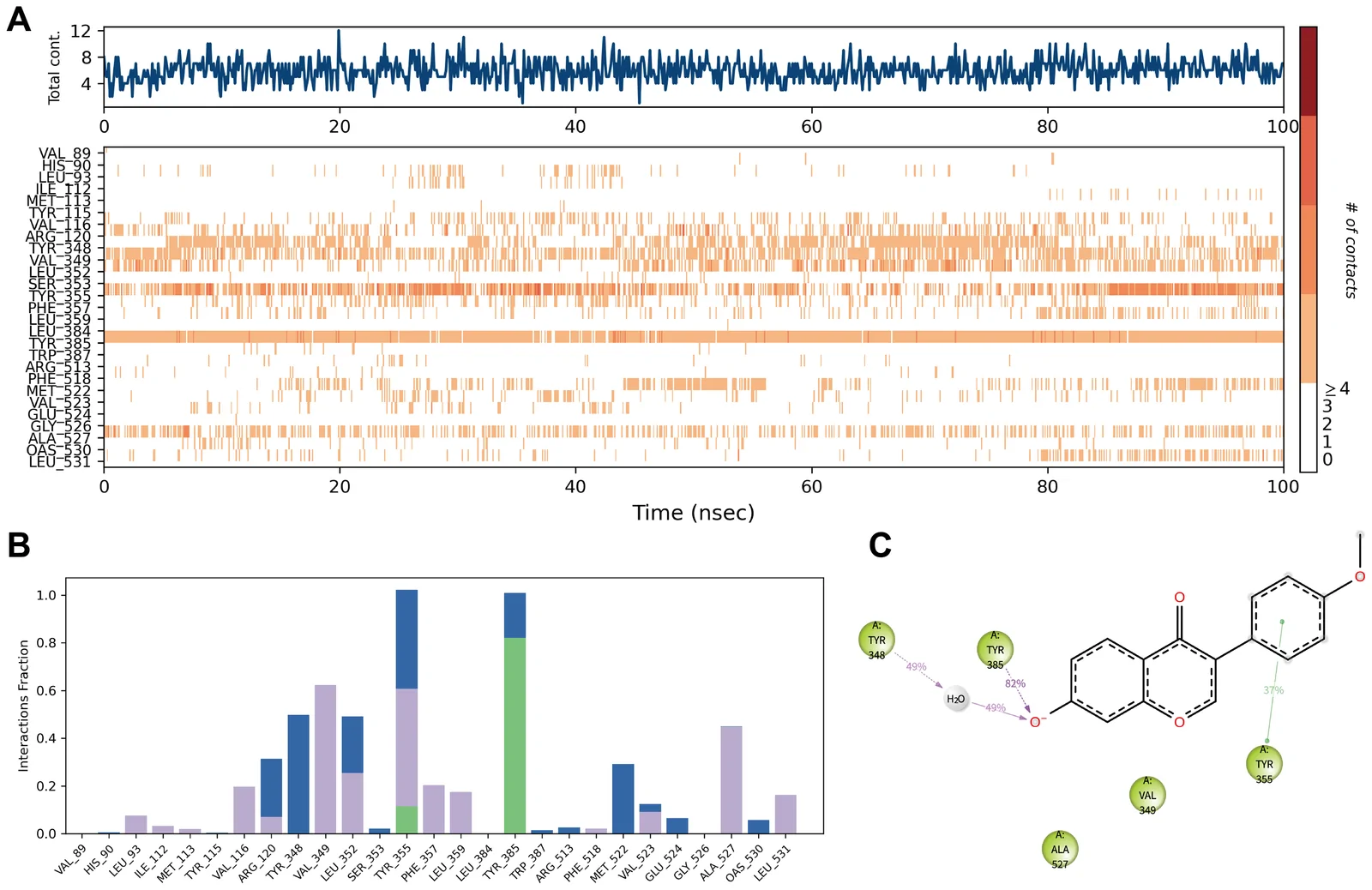
Protein-ligand interaction analysis of the PTGS2-FMNT complex. (A) Timeline representation of interactions. (B) Protein-ligand contact profile. (C) 2D interaction diagram showing interactions occurring in ≥ 30.0% of the simulation time.

The interaction timeline (Fig. 7A) demonstrated that FMNT established sustained contacts with multiple active-site residues throughout the simulation, with hydrogen bonds and hydrophobic contacts being the predominant interaction types. The stacked bar charts (Fig. 7B) show the fraction of simulation time during which specific protein-ligand contacts were maintained. Key residues involved in stabilizing FMNT within the PTGS2 binding pocket included ARG120, TYR355, and SER530 for hydrogen bonds, and VAL349, LEU359, and LEU531 for hydrophobic contacts. Interactions occurring for >30% of the simulation time are highlighted in Fig. 7C. These results indicate that FMNT maintains a stable binding conformation within the PTGS2 active site primarily through a combination of hydrogen bonding and hydrophobic interactions.

#### Binding free energy estimation

To quantitatively evaluate the binding strength of FMNT to PTGS2, MM-GBSA calculations were performed on 50 equally spaced frames from the last 50 ns of each replicate trajectory. The mean binding free energy (ΔG_bind) across three independent replicates was −47.82 ± 1.13 kcal/mol (mean ± standard deviation (SD)), with individual values of −47.59, −49.04, and −46.82 kcal/mol for Runs 1-3, respectively. This value, together with the favorable docking score (−8.94 kcal/mol), supports the thermodynamic favorability of the FMNT-PTGS2 interaction. The difference in magnitude between the docking score and MM-GBSA value is expected, as MM-GBSA provides a more comprehensive estimate of binding free energy that includes solvation and entropic contributions, whereas docking scores represent simplified interaction potentials. The negative ΔG_bind value reflects a favorable binding equilibrium, with the magnitude suggesting that FMNT can form a stable complex with PTGS2 under the simulated conditions.

Energy decomposition analysis (Table 5) revealed that van der Waals interactions (ΔEvdw = −45.23 ± 0.47 kcal/mol) were the dominant contributor to binding, followed by nonpolar solvation contributions (ΔGnonp = −12.82 ± 0.17 kcal/mol) and electrostatic interactions (ΔEele = −4.95 ± 0.94 kcal/mol). The polar solvation term (ΔGsolv GB = 17.81 ± 1.05 kcal/mol) partially offset these favorable contributions, as expected for a binding interface with significant hydrophobic character. The reproducibility of ΔG_bind across the three replicates (SD = 1.13 kcal/mol) further supports the reliability of the predicted binding affinity.

**Table 5:** The combined free energy repeated three times (unit: kcal/mol).

**Complex**	ΔEele	ΔEvdw	ΔGnonp	ΔGsolv GB	ΔGbind
**Replica 1**	−3.94	−45.67	−12.92	17.55	−47.59
**Replica 2**	−5.79	−44.73	−12.62	16.92	−49.04
**Replica 3**	−5.13	−45.29	−12.92	18.97	−46.82
**Mean ± SD**	−4.95 ± 0.94	−45.23 ± 0.47	−12.82 ± 0.17	17.81 ± 1.05	−47.82 ± 1.13

**Notes.**

Δ**G bind ≈**Δ**Ggas +**ΔG solv **ΔG gas =ΔE vdW +ΔE elec,** Δ**G solv=**Δ**Gsolv GB +** **ΔG**_**nonp**_.

#### Ligand conformational properties

**Figure 8:**
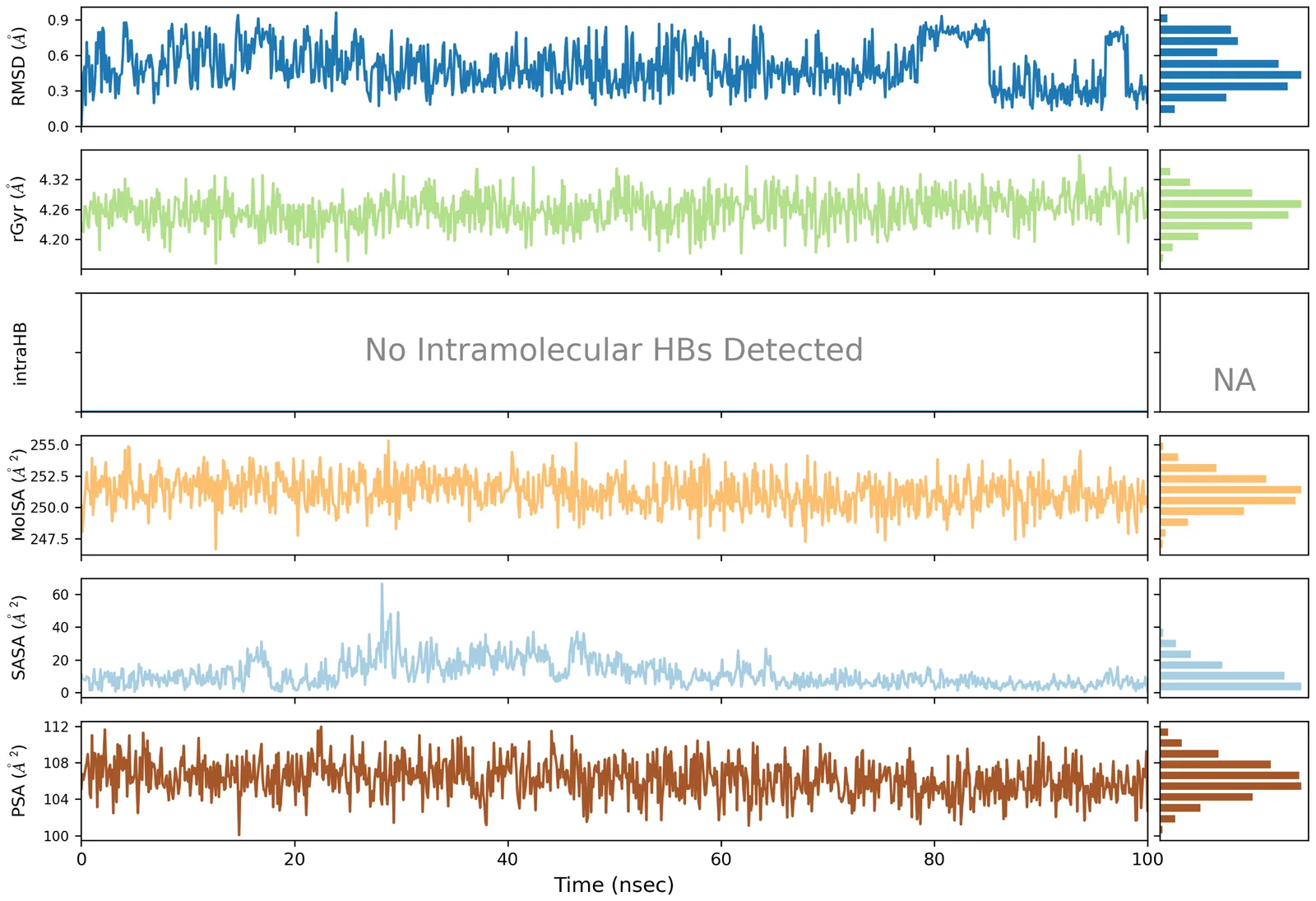
Time evolution of key ligand properties over 100 ns. Ligand RMSD (Å), radius of gyration (rGyr, Å), molecular surface area (MolSA, Å^2^), solvent-accessible surface area (SASA, Å^2^), polar surface area (PSA, Å^2^), and intramolecular hydrogen bonds (intraHB).

The time evolution of ligand properties (ligand RMSD, radius of gyration, molecular surface area, SASA, and polar surface area) is shown in Fig. 8. All parameters remained stable throughout the simulation, indicating that FMNT adopted a compact and minimally deformed conformation within the binding pocket.

## Discussion

By integrating network pharmacology, molecular docking, and MD simulations, we computationally predicted a mechanistic framework for the action of HQ-RS against COPD-associated sarcopenia. Our analysis identified established hub genes (*PTGS2, CDKN1A, NFE2L2, NOS3, MMP2*) and highlighted key signaling pathways, including TNF, IL-17, and AGE-RAGE, which play important roles in inflammation and cellular metabolism. Notably, the central roles of TNF and PTGS2 are consistent with the known pathophysiology of COPD and sarcopenia individually, indicating that our computational model captures established biological processes. To contextualize our findings within the broader literature, we compared them with previous studies. Previous studies have shown that FMNT can alleviate muscle atrophy by modulating pathways such as PI3K/Akt and NF-κB (He et al., 2021; Yoon et al., 2025), which aligns with the inflammatory and oxidative stress pathways enriched in our analysis (*e.g.*, the IL-17 and TNF pathways). However, prior research has largely focused on single diseases or isolated mechanisms. In contrast, our study, through the integration of network pharmacology, computationally predicts which active components of the HQ-RS herbal pair, and through which targets and pathways, can simultaneously target the shared pathogenic mechanisms of COPD and sarcopenia, thereby providing computationally prioritized targets for future investigation of this herbal pair in treating this comorbidity.

At the comorbidity level, TNF-α, as a key regulator of systemic inflammation, has been closely linked to muscle mass loss in COPD patients (Yao et al., 2019; Wu et al., 2023; Kemp et al., 2025; Matera, Page & Cazzola, 2025). Furthermore, the enrichment of pathways such as AGE-RAGE and p53 suggests that HQ-RS may be involved in metabolic stress and cellular senescence—the AGE-RAGE pathway has been implicated in age-related muscle decline (Suzuki, Yabu & Nakamura, 2022; Oh, Jin & Lee, 2023), while the p53 pathway plays a key role in oxidative stress and apoptosis. These pathways likely play important roles in the development and progression of COPD-associated sarcopenia. PTGS2 and its derivative PGE_2_ exhibit a complex role in COPD: on one hand, they contribute to disease progression by promoting inflammation, mucus secretion, and oxidative stress; on the other hand, they may mediate bronchoprotective effects through receptors such as EP4, and their expression is regulated by genetic polymorphisms and multiple signaling pathways (Rumzhum et al., 2016; Martín-Vázquez et al., 2023). However, the exact role of the PTGS2/PGE_2_ pathway in sarcopenia remains controversial. While PGE_2_ may promote muscle breakdown by inducing inflammatory responses, other studies have shown that PGE_2_ is essential for muscle growth and regeneration (Murakami et al., 1997; Brochhausen et al., 2006). This dual role renders the function of this pathway in sarcopenia a complex area of investigation. In the present study, molecular docking and molecular dynamics simulations further indicated that FMNT, an active component of HQ-RS, exhibits favorable predicted binding to PTGS2, a core candidate target of the disease-drug network. MD analysis of the FMNT-PTGS2 complex demonstrated that the system reached dynamic equilibrium after approximately 20 ns, and the ligand maintained a stable binding pose throughout the simulation. RMSF analysis confirmed active-site rigidity, and protein-ligand interaction analysis revealed consistent hydrogen bonds and hydrophobic contacts with key residues. It is important to note that PTGS2 (COX-2) is a promiscuous target that binds a wide range of flavonoid compounds. Therefore, the favorable predicted binding of FMNT to PTGS2 does not necessarily imply selective or physiologically relevant inhibition of PTGS2 in vivo; this requires dedicated experimental validation, including selectivity assays against related enzymes. The FMNT-PTGS2 pair is therefore identified as a computationally predicted compound-target pair warranting further experimental validation. However, the role of PTGS2 (COX-2) in this comorbidity is complex. While COX-2-derived PGE_2_ contributes to COPD pathology by promoting airway inflammation, mucus secretion, and oxidative stress, it also plays a dual role in muscle: PGE_2_ may promote muscle regeneration under certain conditions, yet aberrant activation of the COX-2 pathway in chronic inflammation has been linked to muscle catabolism. This complexity underscores the importance of experimental models that can dissect the tissue-specific and context-dependent effects of FMNT-mediated PTGS2 modulation. Our computational prediction provides a clear molecular entry point for such investigations.

Several limitations of the transcriptomic validation should be acknowledged. The low validation rates (3.3% for COPD and 40.0% for sarcopenia) reflect several inherent challenges in this study. First, the discovery and validation cohorts were derived from different tissue sources (lung *vs.* skeletal muscle), which introduces tissue-specific expression differences. Second, the small sample sizes limit the statistical power for cross-dataset replication. Third, differences in microarray platforms and normalization methods between datasets contribute to technical variability. Despite these challenges, the directional consistency of the 9 shared DEGs across independent datasets provides support for their biological relevance. Future studies with larger, tissue-matched cohorts are needed to validate these candidate genes more rigorously.

Several limitations of the MD simulation analysis should be acknowledged. First, although three independent 100 ns production simulations were performed with different initial velocity seeds, the structural metrics (RMSD, RMSF) across replicates were found to be nearly identical. This was because the Schrödinger Simulation Interaction Diagram (SID) tool, by default, applies an “Average over replicates” function when processing multiple trajectories, which averages the structural metrics across runs. Consequently, replicate-specific variability could not be captured from the SID output. Future studies should export and analyze individual trajectory data for each replicate separately to enable rigorous statistical comparison. Second, the 100 ns timescale is sufficient for evaluating short-term binding stability but may not capture slower conformational rearrangements (Hao et al., 2020). Third, the binding free energy was estimated using MM-GBSA, which, while widely used and computationally efficient, is less rigorous than free energy perturbation (FEP) or thermodynamic integration (TI) approaches (Hou et al., 2011). Nonetheless, the reproducibility of the ΔGbind values across three independent replicates (SD = 1.13 kcal/mol; Table 5) provides confidence in the qualitative conclusion of favorable binding.

Notably, the mechanistic profile revealed by our analysis—specifically, the concurrent modulation of inflammation (*via* TNF and IL-17), cellular metabolism (*via* age-related pathways such as AGE-RAGE and FoxO), oxidative stress (*via* p53 and AGE-RAGE), and smooth muscle proliferation-offers a computational hypothesis for the traditional applications of HQ-RS in treating weakness and debilitating conditions. The potential for additive or cooperative effects of other HQ-RS components on inflammation and metabolism through multiple targets suggests a possible multi-target action that requires experimental confirmation through combination studies. We note that formal synergy modeling or combination analysis was not performed in this study. In addition to the core compound identified, other major components in *Astragalus* and *ginseng* may work together to create a supportive pharmacological microenvironment. For example, astragaloside IV has been shown to improve skeletal muscle mitochondrial function and alleviate oxidative stress (Dai et al., 2023), and ginsenoside Rg1 modulates the immune response and enhances energy metabolism (Wang et al., 2021; Jiang et al., 2024). These components may not directly bind to the core targets identified in this study, but they may exert their effects by ameliorating the systemic inflammatory state, optimizing the tissue microenvironment, or improving the bioavailability of the core active components. This study demonstrates the utility of a reductionist approach to prioritize key effector molecules within complex herbal systems. We also acknowledge that the ultimate efficacy of these molecules *in vivo* is likely to depend on the overall compatibility of the HQ-RS drug pair.

At present, the management strategies for COPD patients mainly focus on symptom control, prevention of acute exacerbations, and improvement in quality of life (Munn et al., 2026). The core treatment methods include the use of bronchodilators, inhaled corticosteroids, and pulmonary rehabilitation. However, these standard therapies have limited effectiveness in improving the systemic inflammation and progressive muscle wasting associated with COPD-related sarcopenia (Bakakos et al., 2025). In this context, traditional herbal medicine is increasingly valued in integrated healthcare models. Recent systematic reviews have begun to recognize the potential value of evidence-based complementary therapies in addressing unmet clinical needs such as fatigue, functional decline, and decreased quality of life (Xiong et al., 2021; Huang et al., 2022; Naas et al., 2026). The findings obtained through computational biology methods in the present study provide a scientific mechanistic hypothesis for how HQ-RS may complement existing standard therapies by simultaneously regulating the inflammatory pathways of the COPD-sarcopenia axis (such as IL-17 and TNF) and stress/metabolic signals (such as AGE-RAGE and p53). Future research should prioritize the experimental validation of these predicted mechanisms in relevant cellular and animal disease models. On this basis, rigorous clinical trials must be designed to evaluate the safety and efficacy of HQ-RS or its refined active ingredients as adjuvant therapy, with a particular focus on COPD patients with early signs of sarcopenia.

Collectively, our integrative computational approach suggests that HQ-RS likely exerts its effects against COPD-associated sarcopenia by simultaneously targeting multiple nodes within a pathological network centered on inflammation and metabolism.

## Conclusions

This study establishes a computational framework that predicts the mechanism of action of the HQ-RS herb pair against COPD-associated sarcopenia. We identified key active compounds (FMNT, kaempferol, 7-O-methylisomucronulatol) and their core targets (PTGS2, CDKN1A, NFE2L2, NOS3, MMP2). Functional enrichment analysis indicates that HQ-RS may concurrently modulate immune-inflammatory pathways (IL-17, TNF), metabolic pathways (AGE-RAGE, PI3K-Akt, FoxO), and stress responses (oxidative stress, p53). Molecular docking and MD simulation with MM-GBSA binding free energy estimation (−47.82 ± 1.13 kcal/mol) across three replicate simulations collectively identify the FMNT-PTGS2 complex as a computationally prioritized candidate for experimental validation. These computational predictions provide a foundation for future targeted drug development and preclinical studies. However, all conclusions remain tentative until experimentally confirmed.

## Supplemental Information

10.7717/peerj.21672/supp-1 Supplemental Information 1 Supplemental figures and tables Fig. S1 Differential expression analysis of COPD.
(A) Volcano plot of DEGs in the COPD discovery set. The x-axis shows log_2_ fold change (log_2_FC) and the y-axis shows –log_2_ P-value. Red dots: significantly upregulated genes (log_2_FC ≥ 1, *P* < 0.05); blue dots: significantly downregulated genes (log_2_FC ≤ –1, *P* < 0.05); grey dots: non-significant genes. (B) Heatmap of the top 50 significantly upregulated DEGs in the COPD discovery set. Rows represent genes, columns represent samples. Expression values are Z-score normalised; red indicates high expression, blue low expression. Hierarchical clustering is applied to both rows and columns. (C) Volcano plot of DEGs in the COPD validation set. Same thresholds and color scheme as in (A). (D) Heatmap of the top 50 significantly downregulated DEGs in the COPD validation set. Presentation and clustering follow the same approach as in (B).
Fig. S2 Differential expression analysis of sarcopenia. (A) Volcano plot of DEGs in the sarcopenia discovery set. Axes and color scheme are the same as in Fig. S1A. (B) Heatmap of the top 50 significantly upregulated DEGs in the sarcopenia discovery set. Normalisation and clustering are identical to Fig. S1B. (C) Volcano plot of DEGs in the sarcopenia validation set. Same thresholds and color code as in (A). (D) Heatmap of the top 50 significantly downregulated DEGs in the sarcopenia validation set. Presentation matches (B).
Fig. S3. Venn diagrams of DEG overlaps and drug–disease target intersection.(A) Venn diagram showing the overlap of DEGs meeting strict thresholds (P ¡ 0.05, —log_2_FC—≥ 1) between the discovery and validation cohorts. No genes were shared between the two sets, indicating an absence of common DEGs under the applied criteria. (B) Integrated evidence network of the 36 final candidate drug–disease targets. Nodes represent candidate targets (green squares) and drugs or disease terms (red inverted triangles). Edges denote supporting evidence from at least one of the following sources: drug targets, shared disease targets, shared DEG evidence, or candidate DEG evidence, thereby providing a visual representation of the multi-source evidence strength for each candidate target.
Table S1.List of 42 active compounds.

10.7717/peerj.21672/supp-2 Supplemental Information 2 Raw data of molecular docking Molecular docking of 15 pairs of compound targets and positive inhibitors.

10.7717/peerj.21672/supp-3 Supplemental Information 3 Molecular Dynamics Simulations of the FMNT-PTGS2 Complex-run 1 The raw data from molecular dynamics simulation 1.

10.7717/peerj.21672/supp-4 Supplemental Information 4 Molecular Dynamics Simulations of the FMNT-PTGS2 Complex-run 2 The raw data from molecular dynamics simulation 2.

10.7717/peerj.21672/supp-5 Supplemental Information 5 Molecular Dynamics Simulations of the FMNT-PTGS2 Complex-run 3 The raw data from molecular dynamics simulation 3.

10.7717/peerj.21672/supp-6 Supplemental Information 6 Documents related to the content of network pharmacology All the files related to the network pharmacology, transcriptome integration and molecular docking analysis of the Huangqi-Shenren drug pair in the intervention of COPD combined with sarcopenia.

## Additional Information and Declarations

### Data Availability

The following information was supplied regarding data availability: The raw measurements are available in the Supplemental Files.
