# Supplementary material for: Active ingredients of Astragalus–ginseng against COPD-associated sarcopenia: network pharmacology and molecular dynamics simulations
Source: PeerJ. 2026 Aug 27;14:e21672. doi: 10.7717/peerj.21672 (PMC13528632; doi:10.7717/peerj.21672)
Supplement: Supplementary file 1 [file peerj-14-21672-s001.zip › Supplementary Materials/Table S1.docx]

Table S1. List of 42 active compounds.

| Compound | OB_percent | DL | Degree | Source_herb |
| --- | --- | --- | --- | --- |
| quercetin | 46.43334812 | 0.27525 | 153 | Astragalus membranaceus |
| kaempferol | 41.88224954 | 0.24066 | 124 | Astragalus membranaceus |
| 7-O-methylisomucronulatol | 74.68613752 | 0.29792 | 45 | Astragalus membranaceus |
| formononetin | 69.67388061 | 0.21202 | 39 | Astragalus membranaceus |
| isorhamnetin | 49.60437705 | 0.306 | 37 | Astragalus membranaceus |
| 3,9-di-O-methylnissolin | 53.74152673 | 0.47573 | 23 | Astragalus membranaceus |
| hederagenin | 36.91390583 | 0.75072 | 23 | Astragalus membranaceus |
| (6aR,11aR)-9,10-dimethoxy-6a,11a-dihydro-6H-benzofurano[3,2-c]chromen-3-ol | 64.25545452 | 0.42486 | 22 | Astragalus membranaceus |
| Calycosin | 47.75182783 | 0.24278 | 22 | Astragalus membranaceus |
| Jaranol | 50.82881677 | 0.29148 | 13 | Astragalus membranaceus |
| Bifendate | 31.09782391 | 0.66553 | 7 | Astragalus membranaceus |
| 1,7-Dihydroxy-3,9-dimethoxy pterocarpene | 39.04541112 | 0.47943 | 4 | Astragalus membranaceus |
| 9,10-dimethoxypterocarpan-3-O-尾-D-glucoside | 36.73668801 | 0.9243 | 3 | Astragalus membranaceus |
| FA | 68.96043622 | 0.7057 | 3 | Astragalus membranaceus |
| (3S,8S,9S,10R,13R,14S,17R)-10,13-dimethyl-17-[(2R,5S)-5-propan-2-yloctan-2-yl]-2,3,4,7,8,9,11,12,14,15,16,17-dodecahydro-1H-cyclopenta[a]phenanthren-3-ol | 36.22847056 | 0.78288 | 1 | Astragalus membranaceus |
| Mairin | 55.37707338 | 0.7761 | 1 | Astragalus membranaceus |
| isomucronulatol-7,2'-di-O-glucosiole | 49.28105539 | 0.62065 | 1 | Astragalus membranaceus |
| (3R)-3-(2-hydroxy-3,4-dimethoxyphenyl)chroman-7-ol | 67.66747949 | 0.26479 | 0 | Astragalus membranaceus |
| 5'-hydroxyiso-muronulatol-2',5'-di-O-glucoside | 41.71766574 | 0.69251 | 0 | Astragalus membranaceus |
| isoflavanone | 109.9866565 | 0.29572 | 0 | Astragalus membranaceus |
| kaempferol | 41.88224954 | 0.24066 | 124 | Ginseng |
| beta-sitosterol | 36.91390583 | 0.75123 | 38 | Ginseng |
| Stigmasterol | 43.82985158 | 0.75665 | 31 | Ginseng |
| Fumarine | 59.26250457763672 | 0.82694 | 28 | Ginseng |
| Inermin | 65.83093145 | 0.53754 | 18 | Ginseng |
| Frutinone A | 65.9037307 | 0.34184 | 16 | Ginseng |
| suchilactone | 57.51882425 | 0.55573 | 16 | Ginseng |
| ginsenoside rh2 | 36.31951162 | 0.55868 | 12 | Ginseng |
| Girinimbin | 61.21530251 | 0.31484 | 10 | Ginseng |
| Aposiopolamine | 66.64690713 | 0.21999 | 8 | Ginseng |
| arachidonate | 45.57324991 | 0.20491 | 4 | Ginseng |
| Dianthramine | 40.44641187 | 0.19676 | 3 | Ginseng |
| Diop | 43.59332547 | 0.39247 | 3 | Ginseng |
| Deoxyharringtonine | 39.27443988 | 0.8116 | 2 | Ginseng |
| Ginsenoside-Rh4_qt | 31.11214828 | 0.77829 | 2 | Ginseng |
| Panaxadiol | 33.0879606 | 0.79404 | 1 | Ginseng |
| alexandrin_qt | 36.91390583 | 0.75268 | 1 | Ginseng |
| Celabenzine | 101.8825954 | 0.48772 | 0 | Ginseng |
| Chrysanthemaxanthin | 38.72398115 | 0.58352 | 0 | Ginseng |
| Gomisin B | 31.99042428 | 0.82858 | 0 | Ginseng |
| ginsenoside Rg5_qt | 39.56307142 | 0.78506 | 0 | Ginseng |
| malkangunin | 57.71384384 | 0.62642 | 0 | Ginseng |
